# Change in faecal incontinence pattern after gastric bypass surgery: related to change in anal sphincter thickness?

**DOI:** 10.1007/s00384-025-05071-w

**Published:** 2026-01-09

**Authors:** Jeff Wennerlund, David Thalén, Anton Östevind, Ulf Gunnarsson, Karin Strigård

**Affiliations:** Department of Diagnostics and Intervention, Surgery, Umeå University, Umeå, Sweden

**Keywords:** Faecal incontinence, Obesity, Anal sphincter, Endoanal ultrasonography, Gastric bypass

## Abstract

**Purpose:**

Faecal incontinence is common in persons with severe obesity. Little is known about how the thicknesses of the internal anal sphincter (IAS) and the external anal sphincter (EAS) change in relation to weight loss following metabolic bariatric surgery (MBS). This study aims to investigate any change in IAS and EAS thickness 6 months after Roux-en-Y gastric bypass surgery (RYGB) and to determine whether any such change correlates with a change in faecal incontinence pattern.

**Methods:**

Thirty-one patients underwent three-dimensional endoanal ultrasound to measure anal sphincter thickness before and 6 months after RYGB. Patients completed the validated Wexner and LARS (low anterior resection syndrome) questionnaires at the same time to evaluate any change in faecal incontinence and urgency symptoms following surgery.

**Results:**

No significant change in the thicknesses of the IAS and EAS was seen. The Wexner score decreased from 18 to 13 (less incontinence). Conversely, the number of patients with LARS increased from 10 to 15 six months after surgery (more urgency).

**Conclusion:**

RYGB had no effect on the thickness of the anal sphincter 6 months after surgery. However, the pattern of faecal incontinence changed, with a decrease in leakage and whole faecal incontinence and an increase in urgency.

## Introduction

Faecal incontinence (FI) is the uncontrolled leakage of stool from the rectum, while anal incontinence is defined as the leakage of either stool or flatus from the rectum. Faecal incontinence is common in patients with obesity but also occurs in the general population [1, 2]. The prevalence of soiling in a Swedish community-based study was 21% in women and 14.5% in men, while the prevalence of solid stool FI was shown to be 1.4% for women and 0.4% for men [2]. In a study by Said, 6.1% men and 5.2% women planned for metabolic bariatric surgery (MBS) had FI. FI has a detrimental effect on the quality of life [3, 4].

The prevalence of FI increases with obesity [1] and increasing age [5]. Women with a body mass index (BMI) over 40 have a three times higher prevalence of anal incontinence than women with a normal BMI [6]. The severity of FI can be decreased by weight loss [7]. MBS is an effective method to induce weight loss and is commonly performed worldwide as the prevalence of obesity increases. MBS is effective in reducing excess weight and in treating and/or preventing obesity-related diseases [8]. Several studies have been carried out to examine whether FI is improved by MBS. Using various validated and unvalidated questionnaires, one review showed that there was a significant reduction in FI in women after MBS [9]. Significant reductions in FI were seen following Roux-en-Y gastric bypass (RYGB) and one anastomosis gastric bypass (OAGB) [9]. Patients lose significant amounts of lean body mass (LBM) and fat-free mass (FFM) after MBS, particularly during the first 3 months, and this continues gradually up to 12 months after surgery [10]. One possible explanation for the decrease in FI after weight loss could be a change in thickness of the anal sphincter, but little is known of the effect of weight loss after MBS on the thickness of the anal sphincter.

Maintenance of continence is a complex mechanism that involves voluntary (external anal sphincter, EAS) and involuntary (internal anal sphincter, IAS) muscle coordination. Maximal anal basal pressure (MABP) is attributed to striated sphincter tonic activity (30%), neural control of IAS activity (45%), purely myogenic IAS activity (10%), and the expansion of the haemorrhoidal plexus (15%) [11]. The IAS decreases in thickness with age along with the resting anal pressure (RAP) [12]. The anterior part of the EAS is absent or decreased in thickness by 50% in women [13], and in healthy individuals increases in thickness with increasing BMI [14].

The aetiology of FI is multifactorial. FI is attributable to conditions associated with pelvic floor weakness and/or diarrhoea [15]. IAS injury and reduced perineal descent are also associated with FI [16]. Independent risk factors for late onset FI in women are smoking, elevated BMI, diarrhoea, irritable bowel syndrome, cholecystectomy, rectocoele, and stress urinary incontinence [17]. Heavy smoking has been shown to be associated with EAS atrophy, as shown by magnetic resonance imaging (MRI) in a case control study [16]. Primiparous women who suffer sphincter injuries during childbirth have a twofold higher risk of developing FI according to a prospective study with a follow-up of 6 months [18].

Three-dimensional endoanal ultrasound (3D-EAUS) is an established method to diagnose sphincter injury and has high sensitivity [19, 20]. Although there have been several studies on FI before and after MBS, no study has used 3D-EAUS to examine sphincter muscle thickness together with the use of validated questionnaires to evaluate FI and urgency symptoms in this patient group.

The hypothesis of this study was that the thickness of the IAS and of the EAS, measured by 3D-EAUS, increases after MBS and that this leads to improvement in FI symptoms assessed by validated questionnaires.

## Material and methods

Patients undergoing RYGB at Lycksele Hospital, located in the Västerbotten Region of northern Sweden, were recruited at the time of their appointment at the surgical department between 2017 and 2022. This was part of the GUMP study, a longitudinal study that measures several physiological and biological outcomes after RYGB. Besides measuring the thickness of the anal sphincter in this study, the larger study includes biopsies and blood samples taken for biochemical markers of muscle and connective tissue, as well as qualitative interview studies. Inclusion was voluntary, and those willing signed an informed consent form. The patient could choose to be included in the GUMP study with or without the anal sphincter investigation. Written and verbal information about the study was given beforehand at an information seminar about MBS (compulsory before surgical consultation). Potential participants were informed that several tests and questionnaires were included and that this required several visits, though some of these could be planned for the same day if that was more convenient for the patient. A total of 49 patients were recruited to the study. Patients were excluded if preoperative data on 3D-EAUS and/or questionnaires were missing.

### Equipment

A 3D-ultrasound apparatus (BK3000™) was used with the anorectal transducer 3D 20R3 (9052) at 13 megahertz (MHz) (BK Medical, GE Healthcare). The images were analysed using BK Medical software BK3D viewer.

### Collection and management of data

3D-EAUS was performed prior to RYGB and 6 months after. At the same time, to assess the incidence and severity of faecal incontinence and associated symptoms, participants were asked to fill out questionnaires on the subject. Currently, there are no specific questionnaires for RYGB patients regarding this; therefore, the participants answered the validated questionnaires Low Anterior Resection Syndrome (LARS) with emphasis on urgency symptoms, in combination with Wexner’s questionnaire, which covers different FI symptoms such as flatus, fluid, and solid incontinence. Both LARS and Wexner’s have been used previously to assess fecal incontinence after obstetric injury [21] and LARS has also been used to explore anorectal dysfunction after cystectomy [22] and surgery for deep endometriosis with and without bowel resection [23].

Wexner’s questionnaire has multiple choice questions on five incontinence problems: (1) incontinence of solid stool; (2) incontinence of fluid stool; (3) incontinence of flatus; (4) need for underwear protection because of incontinence; and (5) lifestyle changes. Each problem is graded from 0 to 4 points, with a possible maximum of 20 points. All scores above zero indicate some degree of incontinence, with increasing scores indicating more problems with incontinence.

LARS questionnaire explores gastrointestinal function with emphasis on urgency symptoms. There are five multiple choice questions with a score for each. The questions are as follows: (1) Is there ever an occasion when you can’t control your flatus (wind)? (2) Do you ever have accidental leakage of liquid stool? (3) How often do you open your bowels? (4) Do you ever have to open your bowels again within one hour of the last bowel opening? (5) Do you ever have such a strong urge to open your bowels that you have to rush to the toilet? A maximum of 42 points is possible. Those without LARS have a score between zero and 20 points. All answered questionnaires were kept in a locked room at the clinic. All data were anonymised, encoded, and then transferred to a Microsoft® Access database.

### Measurement of IAS and EAS

**Fig. 1:**
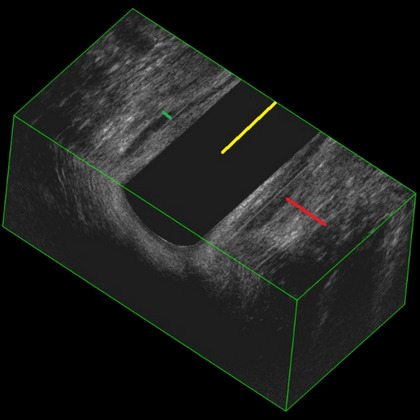
3D-EAUS. Yellow line, halfway through the anal canal. Red line, external anal sphincter. Green line, internal anal sphincter

The 3D image from each examination with the best quality was chosen for evaluation. Possible structural damage, fistula, or other abnormalities were noted. The thicknesses of IAS and EAS were measured halfway along the anal canal and halfway on vertical images (Fig. 1). No rectal ultrasound imaging was performed in the study.

All 3D-EAUS images were procured by DT or KS, anonymised, and stored by a research secretary. Images were then evaluated independently by two of the authors: KS, with long clinical experience of 3D-EAUS, and AÖ, a medical student with an interest in the field. The results were then compared, and any inconsistencies were revisited by both examiners to give a more accurate evaluation, primarily to increase the internal validity of this method. The results were then encoded and entered into the database.

### Database collection

Patient characteristics included preoperative and postoperative appointment dates, gender, date of birth, date of surgery, BMI before and after surgery, diabetes, hypertension, hyperlipidaemia, cardiovascular disease, neuropathy, past pregnancies and gynaecological procedures, previous proctological procedures, and previous radiation therapy. Measurements recorded were anal canal length before and after surgery, signs of previous anal sphincter damage in the best 3D-EAUS picture, IAS thickness in millimeters before and after surgery, and EAS thickness in millimeters before and after surgery. Other relevant information was noted in a free text column. Since this was a part of the GUMP study, no power analysis was done for this part of the trial.

### Statistics

Data were transferred to IBM® SPSS Statistics (IBM Corp. Version 27.0) for statistical analyses. When analysing LARS and Wexner, only the total sums of points were analysed. The Wilcoxon Signed Rank Test was used to compare IAS thickness, EAS thickness, total LARS score, total Wexner score, and BMI before and after surgery. Statistical significance was defined as *p* < 0.05. Spearman’s rho was used to examine correlations between IAS thickness, EAS thickness, total LARS score, total Wexner score, and BMI after surgery.

### Ethics

Ethics approval for the study was obtained from Umeå Regional Ethics Committee 1/12/2016 case number DNR 2015-367−31. The study adhered to the Helsinki Declaration and its amendments.

## Results

**Fig. 2:**
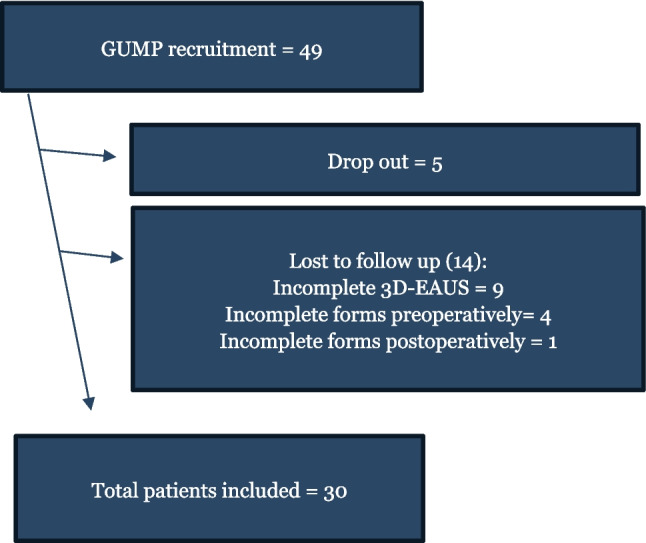
Participant recruitment flowchart

Of the 49 patients available for inclusion, nine patients did not complete both 3D-EAUS examinations, five did not want to participate after surgery, four were excluded due to incomplete preoperative forms, and one patient did not complete the postoperative forms (LARS and Wexner questionnaires). This resulted in 30 included patients who had completed all 3D-EAUS examinations and forms (Fig. 2). Five patients had sphincter disruptions; however, these disruptions did not engage the part of the anal canal which was measured for the thicknesses of IAS and EAS as described previously (Fig. 1). Basic demographics are shown in Table 1.

**Table 1:** Basic demographics. Sphincter Injury = IAS or/and EAS as seen on 3D-EAUS

	All	Women	Men
Number of participants, *n* (%)	30	21 (70%)	9 (30%)
Age, y (min–max)	41 (25–72)	43 (26–72)	40 (25–55)
BMI preop, kg/m (min–max)	41 (34–55)	40.9 (34–55)	41.3 (34–48)
BMI Postop (min–max)	27.6 (21–46)	26.9 (21–46)	29.2 (25–36)
Previous pregnancy, *n* (%)		15 (71%)	
Sphincter injury, *n* (%)	5 (16.6%)	4 (19%)	1 (11%)

### IAS and EAS thickness

**Fig. 3:**
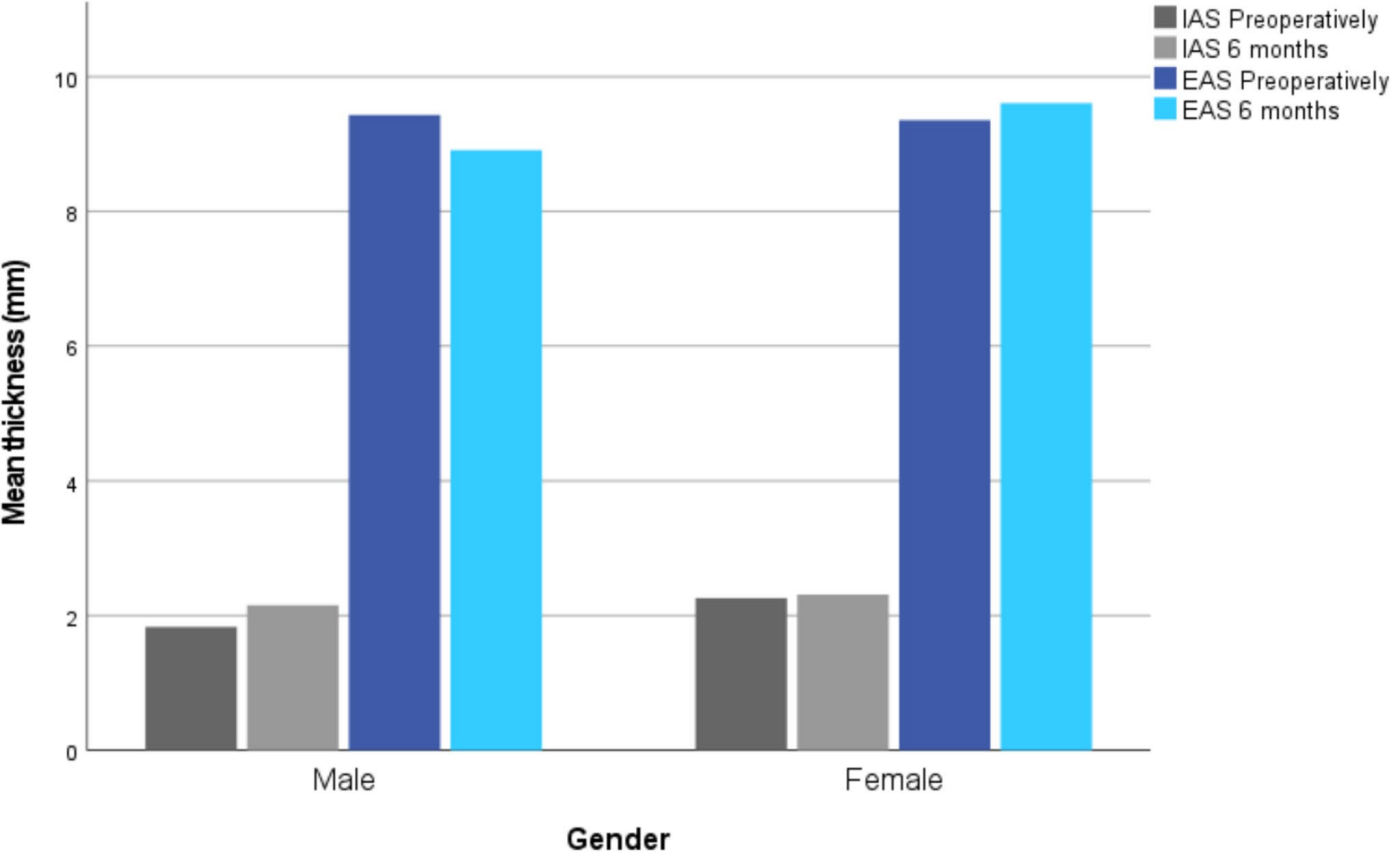
Thickness in mm, IAS and EAS before and 6 months after surgery for men and women. No significant differences was seen

The median IAS thickness was 2.1 mm (1–4) preoperatively and 2.3 mm postoperatively (1–4). Median EAS thickness was preoperatively 9.4 mm (7–16) and postoperatively 9.3 mm (8–15). Comparison of thicknesses before and 6 months after surgery showed no statistically significant differences (IAS *p*-value 0.13, EAS *p*-value 0.78); see Fig. 3.

### Wexner and LARS questionnaire results

There were 18 patients with a Wexner incontinence score (> 0) preoperatively and 13 six months after surgery (Table 2). Ten patients had urgency symptoms according to LARS before surgery and 15 six months after surgery. The Wexner scores (*p*-value 0.89) and LARS scores (*p*-value 0.54) showed no statistically significant difference.

**Table 2:** Results from LARS and Wexner questionnaires prior to and 6 months after gastric bypass surgery. No statistical difference was shown

**LARS score:**	**Preoperative**	**Postoperative**
No LARS, *n* (%)	20 (67)	15 (50)
Minor, *n* (%)	3 (10)	11 (37)
Major, *n* (%)	7 (23)	4 (13)
**Wexner**	**Preoperative**	**Postoperative**
Score: 0, *n* (%)	12 (40)	17 (57)
Over 0, *n* (%)	18 (60)	13 (43)

## Discussion

Weight loss after RYGB had no impact on the thickness of the IAS nor the EAS 6 months after surgery in this study. Despite significant weight loss including lean body mass, the sphincter muscle thickness in the anal canal was not affected. The follow-up time of 6 months was relatively short, and patients could still be in a catabolic state which could have a negative effect on muscle thickness [10] and possibly the function and thickness of the sphincter muscle complex. In a study by Farag et al., 75 patients with haemorrhoids were compared with 75 controls. The controls had a median resting IAS thickness of 2.4 mm (± 0.5 mm) and resting EAS thickness of 5.7 mm (± 0.9 mm) [24], which is consistent with our IAS thickness of 2.1 mm, but our patients had thicker EAS of 9.4 mm. However, a recent study examining patients with obstetric anal sphincter injuries using 3D-EAUS showed a median EAS thickness of 10.4 mm which is more in line with our results [25].

3D-EAUS is an excellent tool to visualise anal muscle structures and pathological features such as a tear in the EAS after childbirth or a fistula. Manometry is used to measure neuromuscular function of the anal canal. It gives information about pressure changes during defaecation contractions and physiological capability [20]. It also provides valuable information about RAP of the anal sphincter and can show if a certain section of the anal canal is dysfunctional [20]. Furthermore, anal electromyography (EMG) and pudendal nerve terminal motor latencies (PNTML) are important tools to diagnose denervation of EAS and muscle weakness leading to faecal incontinence [20]. Although these last-named examinations could have been useful in this project, the thought of so many rectal tests might have resulted in increased difficulty in recruiting patients and a higher drop-out frequency due to the intimate nature of these examinations.

A Swedish community-based study published in 2017, including 268 patients and using questionnaires, showed that there was increased bowel movement, flatus, and urgency symptoms after MBS, but fewer bowel movements after RYGB [26]. This is in line with our results showing increased LARS symptoms of urgency after surgery. On the other hand, we saw a decrease in Wexner incontinence score.

The results found no statistically significant change in LARS and Wexner total scores before and after surgery, but the number of patients with a Wexner score indicating FI decreased, which did not correlate with the unchanged thickness of the EAS. Regarding LARS, the number of patients with urgency increased after surgery despite no change in IAS. LARS and Wexner questionnaires are commonly used in clinical practice to quickly and easily identify patients suffering from FI. However, there is a debate regarding the accuracy and comprehensiveness of these questionnaires [27, 28]. As the aim of this study was to investigate the thicknesses of EAS and IAS pre- and postoperatively together with incontinence scores, and not to assess the direct severity or the appropriate therapy option, we found these questionnaires to be adequate and suitable for the present study. The combination of LARS and Wexner scores has previously been shown to contribute complementary information, although in another study population setting [21].

There are other factors that can affect continence. A recent study by Lodhia et al. examined high-resolution anal manometries of patients with anorectal symptoms, showing that a BMI > 35 was an independent risk factor for FI but also that the mean RAP was higher in this group compared to patients with a BMI < 35 [29]. Those results could imply that IAS and EAS thicknesses remain unchanged 6 months after RYGB, but the mean RAP decreases following weight loss; further studies are required. It remains unclear if weight loss following MBS affects the thickness of the IAS or EAS, and a larger population with a longer follow-up time may be necessary to shed more light.

A change in dietary lifestyle is a necessary part of adjusting to the altered anatomy and physiology after MBS. The amount and frequency of meals and type of food are very different during the first months after MBS compared to before, although by 6 months most patients are eating normal consistency foods in our experience. Previous research from Sweden has found reduced bowel movements after RYGB [26]. An earlier study reported increased frequency of loose stools or diarrhoea and flatulence after RYGB, though the postoperative follow-up was only 4 months [30]. Nevertheless, this could have been a factor leading to the change in incontinence scores seen in this study.

### Strengths and limitations

To our knowledge, this is the first study on the thicknesses of the IAS and EAS before and after MBS using 3D-EAUS. We believe this is an important subject in view of the increased prevalence of anal and faecal incontinence among persons with obesity, and the detrimental effect it has on the quality of life. 

The use of 3D-EAUS to measure IAS and EAS could yield differences between different investigators. In our study, the images were evaluated independently first, and there were only a few cases which had inconsistencies between the examiners. As we revise these few cases with both examiners to give a more accurate evaluation, we believe that our validity was high. A strength of the 3D-EAUS is that it is an objective, common, and sensitive method to visualise anorectal muscles and any possible damage [19, 20]. It is also possible to make a second assessment of the recorded images for accuracy.

## Conclusion

In this study examining the thicknesses of IAS and EAS, no differences were found 6 months after RYGB. However, the pattern of faecal incontinence changed, with symptoms of leakage and whole faecal incontinence decreasing, but urgency increasing. These results suggest that other physiological changes outside the anal sphincter are responsible for our findings; however, further studies on this subject using other modalities could likely shed more light on the complexity of faecal incontinence in obesity.

## Data Availability

Clinical data were collected during the patients’ endoanal ultrasound examinations. The data are stored in the research facility associated with the researchers at the University Hospital of Umeå. Raw data are not publicly available to preserve individuals’ privacy under the European General Data Protection Regulation.
